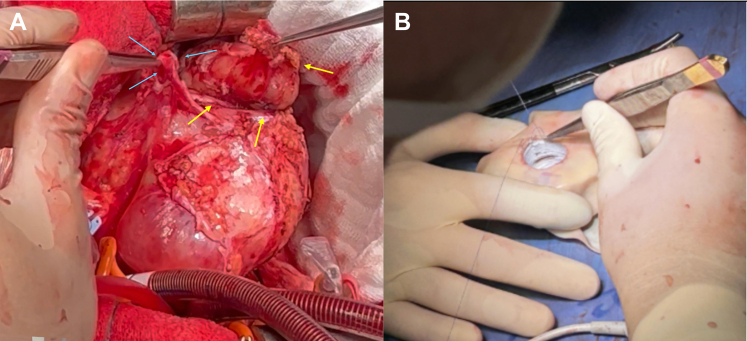# HeartMate 6 in a Total Artificial Heart Configuration After Total Cardiectomy for Cardiac Sarcoma

**DOI:** 10.1016/j.atssr.2025.06.008

**Published:** 2025-07-09

**Authors:** Hrvoje Gasparovic, Maja Cikes, Kristina Krželj, Ivo Planinc, Maja Hrabak Paar

**Affiliations:** 1 University of Zagreb School of Medicine, Department of Cardiac Surgery, University Hospital Center Zagreb, Zagreb, Croatia; 2 University of Zagreb School of Medicine, Department of Cardiovascular Diseases, University Hospital Center Zagreb, Zagreb, Croatia; 3 University of Zagreb School of Medicine, Department of Radiology, University Hospital Center Zagreb, Zagreb, Croatia

## Abstract

We present the case of a 34-year-old patient with an unresectable cardiac sarcoma who underwent a total cardiectomy followed by implantation of 2 HeartMate 3 devices in a total artificial heart configuration. Surgical treatment of cardiac sarcomas ranges from palliative debulking to heart transplantation. In contrast to conventional total artificial heart placement, complete removal of both atrioventricular connections was mandated by the underlying pathologic process. Hemodynamic performance of 2 continuous flow pumps in the absence of native atria and the heart reservoir function depends on balancing preload, afterload, and individual pump rotations. The unreliability of conventional monitoring parameters in this clinical scenario makes hemodynamic management challenging.

The size, location, and infiltrative nature of cardiac sarcomas make them frequently unresectable.^1^^,^^2^ Total cardiectomy followed by cardiac replacement with durable mechanical circulatory assistance may expand the pool of surgically resectable patients with isolated cardiac sarcomas. It can provide prolonged bridge-to-transplantation support, during which time the patient will not be exposed to the risks of immunosuppression.^3^ Moreover, this will allow ample time for residual metastatic disease, if present, to unmask itself.

We present the case of a 34-year-old woman admitted for chest pain to the emergency department. Echocardiography revealed a pericardial effusion with respiratory flow variations across the mitral and tricuspid valves. Notably, she was found to have a 6.7 × 2.3-cm mass infiltrating the right ventricle and compressing its internal diameter (Figure 1; Videos 1 and 2). A biopsy revealed that the mass was consistent with sarcoma. Whereas positron emission tomography/computed tomography ruled out metastatic disease, increased metabolic activity within the pericardium was documented. The tumor was deemed locally unresectable. She was prescribed an Adriamycin (doxorubicin) and ifosfamide (AI) chemotherapy regimen. During this period, the tumor was found to increase in size while remaining localized to the heart on follow-up positron emission tomography/computed tomography. Taking into consideration the poor prognosis of the underlying disease and the absence of widespread dissemination, we decided to perform a total cardiectomy and heart replacement with 2 HeartMate 3 (Abbott Cardiovascular) pumps. Of note, she was evaluated for the Carmat Aeson total artificial heart (TAH), but she failed to meet the eligibility criteria for anatomic compatibility (Supplemental Figure 1). Intraoperatively, we found evidence of disease extending beyond the anatomic boundaries of the right ventricle. There was a discrete palpable mass overlying the left ventricle that was not described on preoperative imaging. Furthermore, a 1-cm nodule of unclear cause was found between the aorta and the pulmonary artery. The native heart was removed in its entirety, save for a remnant of left atrial tissue surrounding the pulmonary veins (Figure 2A). Although it was appreciated that removal of both atrioventricular connections would make device implantations cumbersome, it was deemed necessary to achieve radical tumor resection. The inflow ring was placed on top of a Teflon sleeve, which was created by connecting multiple layers of Teflon felt with BioGlue (Artivion; Figure 2). These had a central aperture that matched the inflow cannula diameter. The Teflon sleeve was 3 mm shorter than the inflow cannula. The purpose of this maneuver was to distance the cannula tip from the native atrial tissue, thereby preventing suction events. The atrial side of the fenestrated Teflon was covered with a glutaraldehyde-preserved pericardial patch to reduce its thrombogenic potential (Figure 2A; Supplemental Figure 2B). The native left atrial remnant was then anastomosed to the Teflon-enforced inflow ring. A neo–right atrium was created by bridging the gap between the superior vena cava and inferior vena cava with a 32-mm Dacron graft (Figure 2B). An aperture matching the external diameter of the inflow cannula of the future HeartMate 3 right ventricular assist device (RVAD) was created. Another Teflon sleeve for the inflow ring was created. The outflow grafts were then connected to the aortic and pulmonary artery stumps in an end-to-end fashion (Figure 3). The mismatch between the outflow grafts and the native aorta and pulmonary artery was not prohibitive for a direct end-to-end anastomosis. Both pumps were initially started at minimal speeds of 3000 rpm. The pump rotations were slowly increased while maintaining a lower RVAD speed than on the left side. Pump rotations were calibrated to equalize the left ventricular assist device (LVAD) and RVAD flows while reducing cardiopulmonary bypass flows. Close attention was paid to avoidance of pulmonary overcirculation throughout the early postoperative course. The patient’s initial ventricular assist device settings were as follows: LVAD 4900 rpm, flow 3.0 L/min; RVAD 4600 rpm, flow 3.0 L/min, which provided 80 mm Hg of mean arterial pressure. Introduction of vasodilators and titration of mean arterial pressure led to an increase in effective flows (LVAD from 3.6 to 4.2 L/min, RVAD from 3.3 to 4.1 L/min) without changing pump speeds. Sternal closure was delayed for a day to ensure that a quick surgical response was available if the achieved equilibrium between the 2 pump flows became compromised.

**Figure 1:**
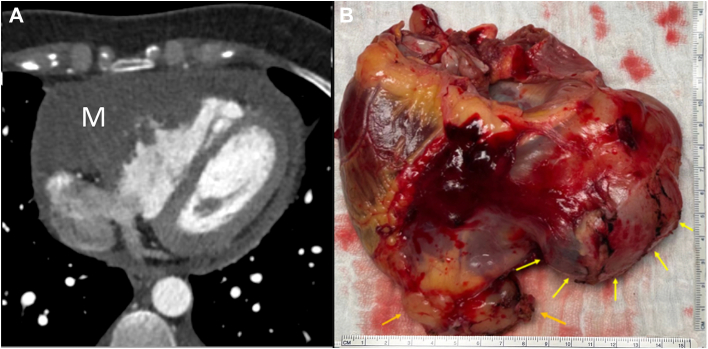
(A) Cardiac computed tomography showing an intrapericardial mass (M) with malignant imaging characteristics. (B) Macroscopic specimen of the heart showing a large multilobular tumor (arrows).

**Figure 2:**
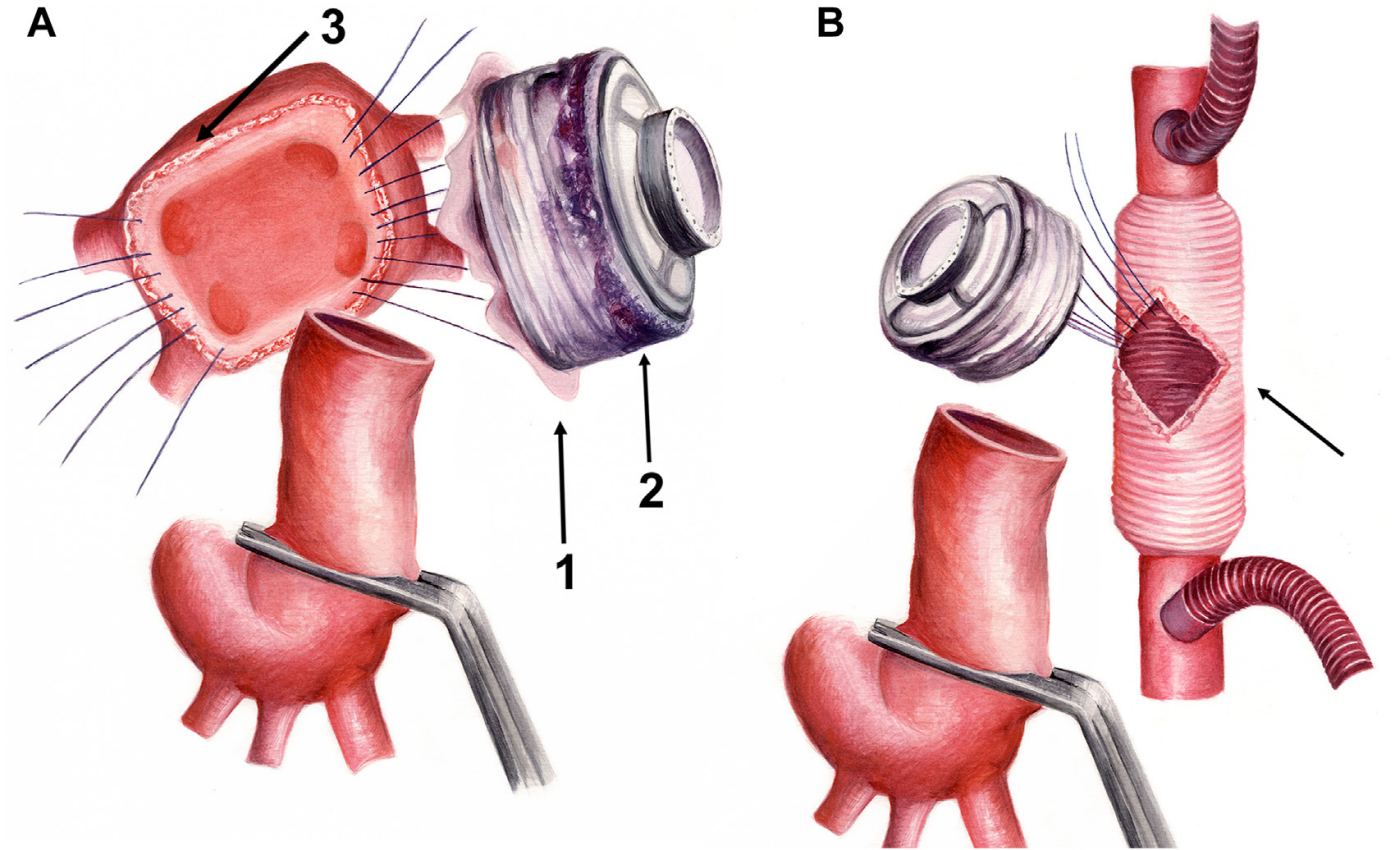
(A) Left atrial HeartMate 3 left ventricular assist device placement (1, pericardial cover; 2, Teflon sleeve; 3, left atrial remnant). (B) Right atrial HeartMate 3 right ventricular assist device placement (arrow, Dacron graft bridging the gap between the superior vena cava and the inferior vena cava with an aperture matching the inflow ring internal diameter).

**Figure 3:**
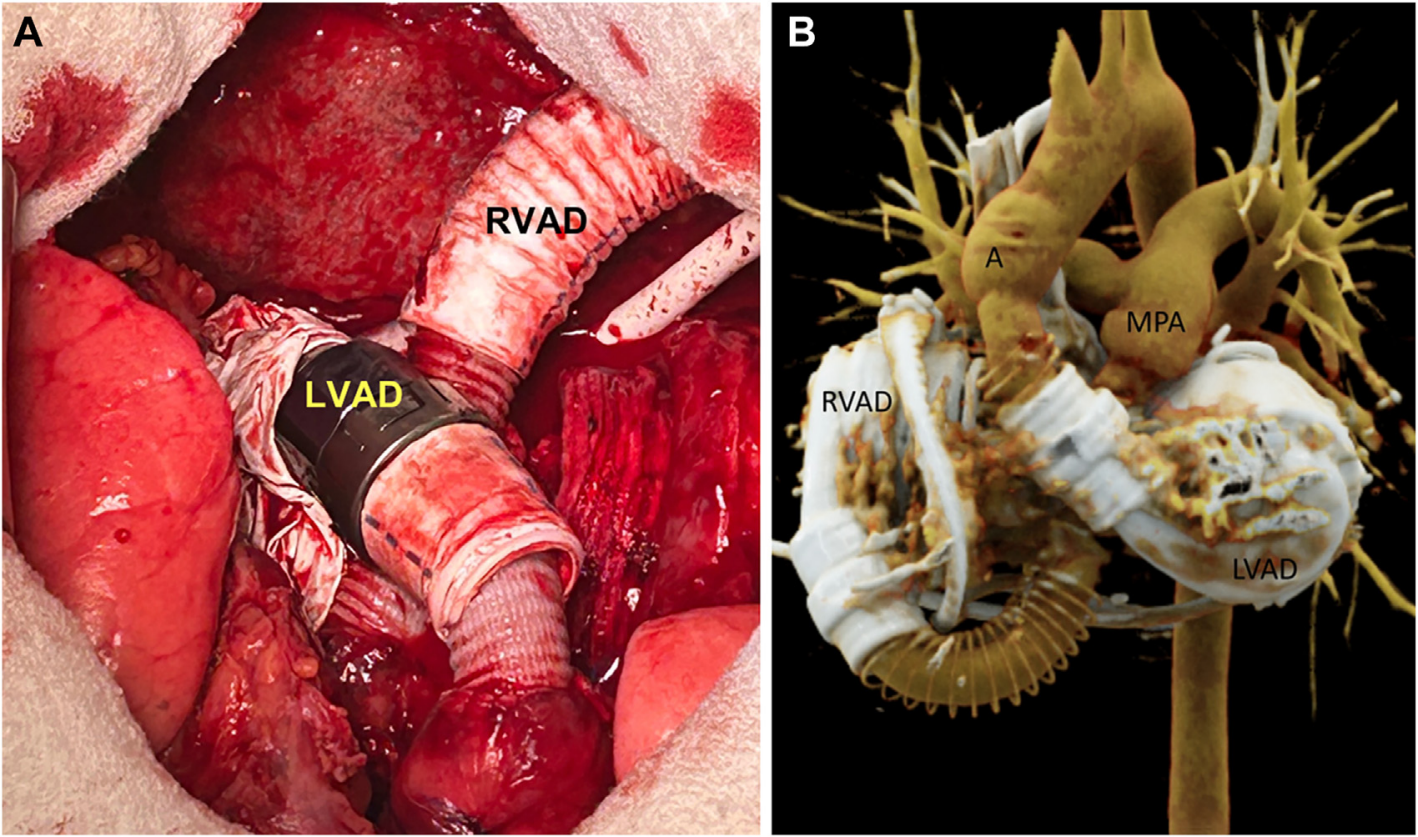
(A) Intraoperative view of the total artificial heart made of 2 centrifugal pumps. (B) Three-dimensional computed tomography reconstruction of the total artificial heart. (A, aorta; LVAD, left ventricular assist device; MPA, main pulmonary artery; RVAD, right ventricular assist device.)

We were concerned that malposition of the intersecting outflow grafts and 2 intrapericardial pumps, seated in nonsolid tissues, might destabilize hemodynamics. Before chest closure, we inserted a breast implant into the pericardial cavity to preserve space for a future heart transplant, should the patient ever become eligible. The histologic specimen revealed extensive disease. In addition to an 8 × 4.5 × 9-cm myxoid pleomorphic liposarcoma, 2 secondary epicardial tumors overlying the left ventricle were noted. At 4 months postoperatively, our patient had resumed normal daily activities. Her latest ventricular assist device settings were 4900 and 4400 rpm, providing flows of 4.2 L/min and 3.8 L/min for her LVAD and RVAD, respectively.

## Comment

We have demonstrated the feasibility of HeartMate 6 implantation in a TAH configuration after total cardiectomy. Our surgical strategy differed from conventional TAH implantation techniques for nonmalignant indications, for which biventriculectomies rather than a total cardiectomy are performed.^4^ The advantage of biventriculectomies is that muscular ridges distal to both atrioventricular grooves are preserved, which facilitates pump positioning and stabilization. In addition, the native atrial volumes provide residual cardiac reservoir function. Removal of the entire heart renders the patient susceptible to abrupt and potentially fatal hemodynamic disturbances related to pump destabilization or interference with the contralateral device. Preventing catastrophic suction events is paramount in this clinical setting, with little margin for error. Meticulous care was therefore taken in creating enough space between the tips of both inflow cannulas and the adjacent tissues. Notwithstanding the absence of native atrial reservoirs, the patient’s pump flows were stable and sufficient for the resumption of normal daily activities. We recognize that the excellent outcomes of isolated LVADs have not been duplicated in the biventricular failure setting.^5^ Both biventricular assist devices and TAHs have higher mortality and morbidity burdens. Future heart transplantation therefore remains an optimistic strategic goal for our patient, conditional on the absence of tumor recurrence.

## Figures and Tables

**Supplementary Figure 1:**
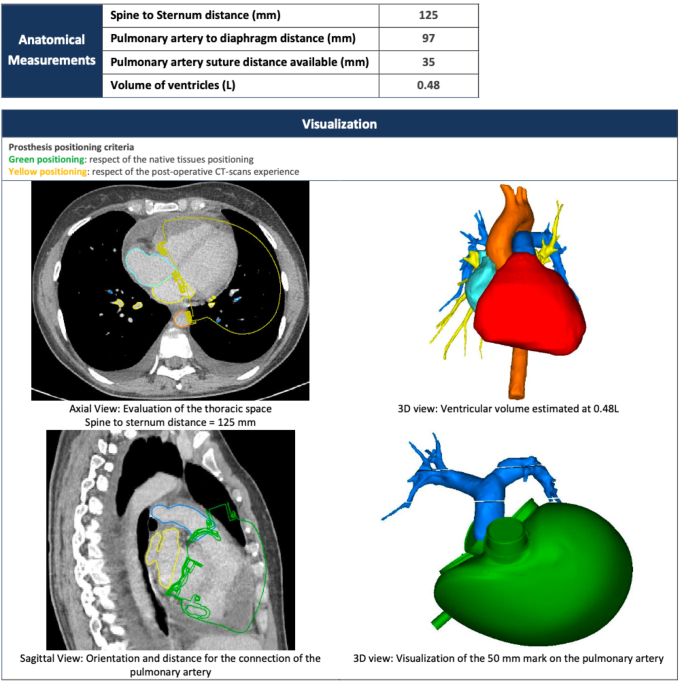


**Supplementary Figure 2:**