# Supplementary material for: HeartMate 6 in a Total Artificial Heart Configuration After Total Cardiectomy for Cardiac Sarcoma
Source: Ann Thorac Surg Short Rep. 2025 Jul 9;3(4):1116–9. doi: 10.1016/j.atssr.2025.06.008 (PMC12712172; doi:10.1016/j.atssr.2025.06.008)
Supplement: Supplementary Figures legends [file mmc1.docx]

**SUPPLEMENTAL FIGURE LEGENDS**

**Supplemental Figure 1.** CARMAT Virtual Implantation Report illustrating the outlines of the device within the chest cavity and its potential to compress native vascular structures.

**Supplemental Figure 2.** A. Macroscopic specimen of the heart showing the primary tumor (yellow arrows) and a subepicardial mass of unknown etiology (blue arrows). B. Back-table pump preparation with suturing of a pericardial patch to cover the teflon sleeve.
